# The role of the hippocampus in weighting expectations during inference under uncertainty

**DOI:** 10.1016/j.cortex.2019.01.005

**Published:** 2019-06

**Authors:** Francesco Rigoli, Jochen Michely, Karl J. Friston, Raymond J. Dolan

**Affiliations:** a City, University of London, London, UK; b The Wellcome Trust Centre for Neuroimaging, UCL, London, UK; c Max Planck UCL Centre for Computational Psychiatry and Ageing Research, London, UK

**Keywords:** Hippocampus, Inference, Bayesian, Prior, Uncertainty

## Abstract

Making inference under uncertainty requires an optimal weighting of prior expectations and observations. How this weighting is realized in the brain remains elusive. To investigate this, we recorded functional neuroimaging data while participants estimated a number based on noisy observations. Crucially, the prior expectation about the variability of observations (an expected variability) was manipulated. Consistent with normative models, when novel observations were characterized by higher expected or observed variability, participants' estimates relied more on expectations than novel observations and were characterized by higher stochasticity. Activity in hippocampus increased when novel evidence was characterized by higher expected or observed variability. Response in superior parietal cortex reflected a precision-weighted prediction error signal (i.e., the distance between observations and expectations) that was modulated by hippocampal activity. Our findings implicate the hippocampus during inference under uncertainty, suggesting a role in weighting prior representations over observations and in modulating responsivity of superior parietal cortex to prediction error.

## 1 Introduction

In daily life, we often face situations that require inference based on ambiguous or noisy sensory data, a form of inference under uncertainty (Chater et al., 2006, Clark, 2013a, Clark, 2013b, Friston, 2010, Hohwy, 2013, Vilares and Kording, 2011). A paradigmatic example is driving a car in the fog, which requires veridical inference about key states of affairs – such as the trajectory of the road or inferred speed of the vehicle – from a noisy or imprecise visual input. A key aspect of such inference under uncertainty is an integration of prior knowledge and incoming sensory evidence. During estimation of a continuous variable from noisy observations, different forms of prior information can be considered. One of these is expected value, which is associated with a prior uncertainty reflecting confidence in an expectation. Another is the expected variability of upcoming sensory evidence. For example, if we need to infer how buildings in a city will vary in size based on data derived from one particular area, knowledge of similar cities can inform prior beliefs on such variability. This *expected variability* can then be integrated with data, or *observed variability*, to estimate a posterior belief about the buildings' variability.

Prior studies have primarily focused on the manipulation of expected value and its uncertainty, where an influential body of work proposes these quantities are treated in a manner consistent with optimal (or Bayesian) inference (Chater et al., 2006, Clark, 2013a, Clark, 2013b, Friston, 2010, Hohwy, 2013, Vilares and Kording, 2011). Substantial empirical evidence now supports this notion (Büchel et al., 2014, Berniker and Kording, 2008, Ernst and Banks, 2002, Harris and Wolpert, 1998, Kersten et al., 2004, Knill and Richards, 1996, Kording and Wolpert, 2004, Körding and Wolpert, 2006, Jazayeri and Shadlen, 2010, Petzschner et al., 2015, Summerfield and De Lange, 2014, Summerfield and Egner, 2009, Trommershauser et al., 2011, Trommershäuser et al., 2003, Trommershäuser et al., 2008, Todorov, 2004, Wolpert et al., 1995, Zelano et al., 2011). However, the role of prior expectations regarding variability in upcoming sensory data remains poorly understood and it remains unclear how the brain processes expectations about variability during inference.

Here, we investigated the integration of expected and observed variability during inference by characterising the associated cognitive and neural processes. We devised a new task where participants are asked to infer the value of a number based on both prior information and noisy observations. To test key predictions of an optimal inference hypothesis, we manipulated (i) the expected value of the number, (ii) the expected variability of observations, and (iii) the actual variability of observations. This enabled us to examine the influence of *expected* and *observed* variability on an estimation of the number. Theoretical models of optimal inference predict that observations with high expected or observed variability should be considered as less reliable (Chater et al., 2006, Clark, 2013a, Clark, 2013b, Friston, 2010, Hohwy, 2013, Vilares and Kording, 2011). Hence, with less reliable observations, the number estimated by participants should be closer to the expected value than to the value indicated by a novel observation. Additionally, less reliable observations should also increase response stochasticity, i.e., the variability of participants' estimates.

Using functional neuroimaging, we recorded participants' brain activity during task performance to elucidate important aspects of inference that remain poorly understood. We asked how the brain realizes a weighting of expectations over observations, which prescribes how much one should rely on prior information compared to upcoming and novel sensory evidence. Specifically, a region involved in weighting expectations over observations was predicted to show enhanced activity for both higher expected and observed variability. In addition, we also examined how the brain represents prediction error (PE; i.e., the distance between observations and expectations), which is another important quantity that guides inference. Finally, we explored the relationship between neural processes linked with weighting expectations over observations and neural processes linked with PE signalling. As suggested by some theoretical proposals (Friston, 2005, Rao and Ballard, 1999), a possibility is that regions weighting expectations over observations would modulate activity in regions reflecting PE.

## 2 Methods

### 2.1 Participants

Thirty-three healthy right-handed adults (18 females and 15 males, aged 20–40, mean age 27) participated in the experiment. All participants had normal or corrected-to-normal vision. None had history of head injury, a diagnosis of any neurological or psychiatric condition, or was currently on medication affecting the central nervous system. The study was approved by the University College of London Research Ethics Committee. All participants provided written informed consent and were paid £40 for participating.

### 2.2 Experimental paradigm and procedure

**Fig. 1:**
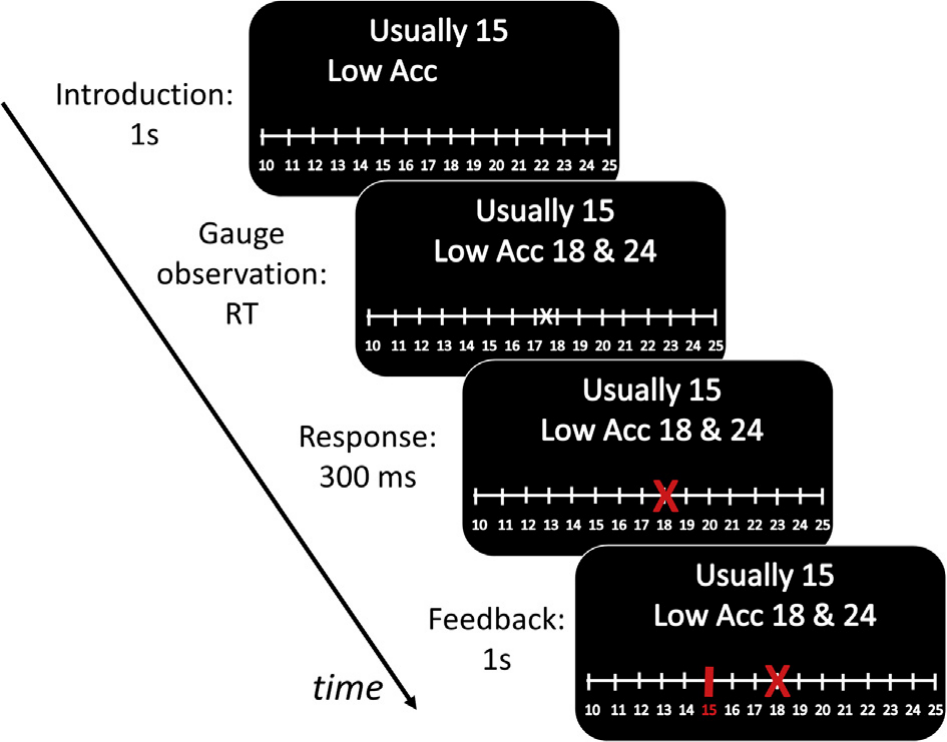
Illustration of the task paradigm. Participants estimated the amount of fuel present in the tank of a motorbike by reporting a number between 10 and 25 L. Participants were told that motorbikes were equipped with two gauges, each providing an independent reading of the fuel amount. For one second, information was provided on the top of the computer screen about (i) the amount of fuel usually present in the motorbike tank (either 15 or 20 L), (ii) the expected variability of the gauges (either low or high). The latter was described to participants as the accuracy of the gauges (Acc), with high accuracy (High Acc) corresponding to low expected variability, and low accuracy (Low Acc) corresponding to high expected variability. Next, two numbers (e.g., 18 and 24) were presented, each indicating the fuel reported by one gauge. At this time, participants could indicate their inferred fuel amount (e.g., 18), and 300 msec after choice feedback on the true fuel amount (e.g., 15) was provided for one second.

During MRI, participants performed a computer-based task lasting approximately 40 min (Fig. 1), which required estimating the value of numbers based on prior information and on noisy observations. The task was based on a cover story, whereby participants estimated the amount of fuel in the tank of a motorbike by reporting a number between 10 and 25 L. Participants were instructed that motorbikes were equipped with two gauges, each providing an independent reading of the fuel amount. On each trial (there were 480 trials overall), participants observed the numbers reported by the gauges ($g_{1}$ and $g_{2}$, both between 10 and 25 L). Before these numbers appeared, information was provided on the top of the computer screen about (i) the amount of fuel usually present in the motorbike tank (either 15 or 20 L), corresponding to an *expected value*, and (ii) the usual variability of the gauges (either low or high), corresponding to *expected variability*. The latter was described to participants as the accuracy of the gauges, with high accuracy corresponding to low expected variability, and low accuracy corresponding to high expected variability. One second after presentation of prior information, the two numbers $g_{1}$and $g_{2}$ were presented. These were characterized by an *observed variability*, in other words numbers that were very close together resulted in a low observed variability, while numbers that were far apart were indicative of a high observed variability.

The prior information (expected value and expected variability) given to participants was reliable, with the true fuel amount selected randomly from a distribution with an average corresponding to the expected value, and where the distance between $g_{1}$ and $g_{2}$ was on average larger for trials with high compared to low expected variability. Specifically, for each trial the true fuel amount *μ* was randomly drawn from a Gaussian distribution with mean equal to either 15 or 20 (i.e., the expected value), and SD equal to 3. The quantities reported by the gauges corresponded to two numbers $g_{1}$ and $g_{2}$ independently drawn from a Gaussian distribution with mean *μ* and SD equal to 4 during low expected variability trials, and equal to 7 during high expected variability trials. The values of *μ*, $g_{1}$ or $g_{2}$ were rounded to the nearest integers, and if one of them was larger than 25 or smaller than 10, it was assigned the closest between 25 and 10.

After the numbers reported by the gauges $g_{1}$ and $g_{2}$ appeared, participants could indicate their inferred fuel amount by selecting a number between 10 and 25 using a keypad to move a cursor on a scale. The keypad included one button for moving the cursor left and another button for moving the cursor right, plus a button to finalize the choice. 300 msec after the choice was finalized, feedback on the true fuel amount was provided, as the corresponding number on the scale turned red for one second, and a new trial started immediately after.

A new motorbike was presented on each trial. However, to facilitate processing of prior information, the task was organized in blocks, each with 5 consecutive trials presenting motorbikes characterized by the same expected fuel amount and the same expected variability level. Before a new block started, the statement “New set” appeared for two seconds. Block order was pseudo-random, and an equal number of trials was presented for each condition of usual fuel amount and of expected variability.

Participants were tested at the Wellcome Trust Centre for Neuroimaging at the University College London. Before scanning, they were fully instructed about the task and played 120 trials, ensuring they familiarized with task statistics. This was aimed at minimizing any influence of learning, hence isolating the computational and neural processes underlying inference. This allowed us to focus specifically on how the brain realizes inference based on prior knowledge which has been previously acquired through learning. Inside the scanner, participants performed the task in four separate sessions (each including 120 trials), followed by a 12 min structural scan. After scanning, participants were debriefed and received a remuneration of £40 for participating.

### 2.3 fMRI scanning and analysis

The task was programmed using the Cogent toolbox (Wellcome Trust Centre for Neuroimaging) in Matlab. Visual stimuli were back projected onto a translucent screen positioned behind the bore of the magnet and viewed via an angled mirror. Blood oxygenation level dependent (BOLD) contrast functional images were acquired with echo-planar T2*-weighted (EPI) imaging using a Siemens Trio 3-Tesla MR system with a 32 channel head coil. The whole brain was covered by images comprising 48 interleaved 3-mm-thick sagittal slices (in-plane resolution = 3 × 3 mm; time to echo = 30 msec; repetition time = 3.36 sec). The first six volumes were discarded to allow for T1 equilibration effects. T1-weighted structural images were acquired at a 1 × 1 × 1 mm resolution. Functional MRI data were analyzed using Statistical Parametric Mapping (SPM) version 12 (Wellcome Trust Centre for Neuroimaging). Data preprocessing included spatial realignment, unwarping using individual field maps, slice timing correction, normalization and smoothing. Specifically, functional volumes were realigned to the mean volume, were spatially normalized to the standard Montreal Neurological Institute (MNI) template with a 3 × 3 × 3 voxel size, and were smoothed with 8 mm Gaussian kernel. High-pass filtering with a cut-off of 128 sec and AR(1)-model were applied.

We characterised the neural processes underlying the weighting of prior expectations and observations during inference. Specifically, we probed brain activity as a function of expected and observed variability, and in relation to expression of a PE. Hemodynamic responses were modelled with a canonical hemodynamic response function and a GLM including, when the two numbers indicated by the gauges $g_{1}$ and $g_{2}$ were presented, one stick function regressor for high expected variability trials and another stick function regressor for low expected variability trials. Each was modulated (i) by the PE signal equal to $PE=|\bar{\mu }-\mu_{g}|$, namely the distance between the prior mean $\bar{\mu }$ (either 15 or 20 L) and the observation mean $\mu_{g}$ (corresponding to $\mu_{g}=\left(g_{1}+g_{2}\right)/2$), (ii) by the observed variability $v_{g}$ equal to $v_{g}=|g_{1}-g_{2}|$, namely the distance between the numbers indicated by the gauges, and (iii) by the RT associated with the participant's response measured from the gauge onset as nuisance parametric modulator. For the GLM estimation, the parametric regressors were mean-rescaled except for observed variability. The latter variable was not demeaned for the following reason. Mathematically, a stick function regressor (such as the one for high expected variability or the one for low expected variability) reflects the predicted response when its associated parametric modulators (e.g., PE and observed variability) are equal to zero. By design, high and low expected variability trials were matched with respect to PE, but they were not matched with respect to observed variability. This because, by design, high expected variability trials were, on average, associated with higher observed variability. Therefore, if in the GLM the observed variability was rescaled to the mean (separately for high expected variability and low expected variability), then, when comparing high versus low expected variability trials, the same rescaled observed variability levels would correspond to different raw observed variability levels. A consequence of this would be a bias when comparing high versus low expected variability. This bias can be avoided by considering the raw, and not the demeaned, observed variability, an approach we followed in our GLM.

The GLM included other regressors; specifically (i) one stick function regressor at feedback time modulated by the distance between the feedback number and the number chosen by the participant, (ii) a box-car function regressor at the time when the first button of the keypad was pressed, with a duration defined by the time when the response was finalized, (iii) 6 movement and 17 physiological (derived from breathing and heart rate signals) nuisance regressors. The GLM was estimated separately for each session of the task (see Table S1 for information about the collinearity among regressors of the GLM, showing that there are no issues of collinearity in the GLM).

Contrasts of interest were computed subject by subject, and used for second-level (between subjects) one-sample *t*-tests using standard summary statistic approach (Holmes & Friston, 1998). To establish which brain region to focus on for exploring how expectations are weighted over observations, we considered two criteria. First, activation in a region reflecting the weighting of expectations over observations should increase when novel evidence is less reliable, corresponding in our task to trials having higher expected or observed variability. Second, we were interested in regions potentially recruited when abstract quantities are involved, and for this purpose we adopted a task in which an abstract variable was manipulated. Given these two criteria (i.e., the predicted neural activation and the focus on an abstract task), we investigated the weighting of expectations over observations focusing on the hippocampus, for the following reasons. First, it has been shown that activity in this region is sensitive to the entropy of a stimulus stream (Harrison et al., 2006, Strange et al., 2005, Tobia et al., 2012), which is analogous to observed variability in our task. This raises the question of whether response in hippocampus increases also for expected, in addition to observed, variability, as implicated by an encoding of a weight of expectations over observations. Second, a large body of evidence indicates that hippocampal engagement is not bound to any specific sensory modality, and occurs when abstract variables are involved (Hasselmo and Wyble, 1997, McNaughton and Nadel, 1990, Rolls and Treves, 1998). This is in line with the possibility that this region could play a role in our abstract task. Third, although previous evidence indicates that observed variability of novel evidence affects activity also in other regions such as the occipital cortex (Vilares, Howard, Fernandes, Gottfried, & Kording, 2012), these are sensory-specific areas which are less likely to be recruited when abstract quantities are manipulated. For these reasons, we focused on the hippocampus as a candidate structure for encoding the weight of expectations over observations during an abstract task.

Regarding the question of how PE is represented in the brain, evidence from neuroimaging studies (Strange et al., 2005, O'Reilly, Schüffelgen, et al., 2013, O'Reilly, Jbabdi, et al., 2013), as well as a recent computational model (O'Reilly, Schüffelgen, et al., 2013), proposes that the superior parietal cortex (SPC) is critical for processing surprise (indicating how much a new observation is informative). When a continuous variable is manipulated such as in our task, surprise is mathematically equivalent to a precision-weighted PE, in other words to a PE multiplied by its precision (the precision of a variable is the inverse of its variance or uncertainty; see below). This raises the question of whether a precision-weighted PE is signalled within SPC.

For these reasons, statistical (small volume corrected – SVC) tests focused on the hippocampus and the SPC as pre-defined ROIs for the group. For hippocampus, we relied on the pre-defined hippocampal anatomical mask available in the AAL structural ROI archive provided by the MarsBar toolbox (for details on how this mask was derived, see Tzourio-Mazoyer et al., 2002). Previous literature indicates the anterior hippocampal portion is particularly involved in novelty processing. Given our specific interest in this portion, we split the hippocampal mask relative to the vertical axis and we included only voxels with z < −14 in our final hippocampal ROI. The specific portion of the SPC which have been linked with processing surprise (O'Reilly, Jbabdi, et al., 2013) has been labelled as area IPS3 (Mars et al., 2011) or area 7 A (Scheperjans et al., 2008). Similar to O'Reilly, Jbabdi, et al. (2013), our ROI corresponded to an 8 mm sphere centred on *a priori* coordinates extracted from a recent diffusion-imaging parcellation study on this portion of SPC (Mars et al., 2011; ±15, −63, 53). Statistics of ROIs were SVC using a family wise error (FWE) rate of *p* < .05 as the significance threshold. For exploratory purposes, we also report data for other brain regions with statistics having *p* < .001 uncorrected significance (Table S2).

## 3 Results

### 3.1 Behaviour

We analysed how participants inferred the fuel amount and asked whether this was consistent with predictions derived from optimal inference (for additional analyses of reaction times (RTs) see SI). A first prediction is that the higher the expected variability, the closer subjects' estimates should be to the expected value, relative to the mean of the gauges, i.e., the average observed value. Second, when gauges report numbers that are far from each other, thereby increasing observed variability, subjects' estimates should be closer to the expected value relative to the observation mean (and *vice versa* when gauge numbers are close to each other). Finally, we tested implications of optimal inference for the stochasticity of participants' estimates. Specifically, we asked whether the degree of stochasticity (i.e. response variability) remained constant or – as predicted by optimal inference – it increased with both expected and observed variability.

First, we estimated a multiple regression model to assess whether expected and observed variability influence the position of participant's response *R* relative to the expected value $\bar{\mu }$ (either 15 or 20 L) and to the observed mean of the gauges $\mu_{g}$(equal to $\mu_{g}=\left(g_{1}+g_{2}\right)/2$). As dependent variable of the regression model, we considered y = |R − $\mu_{g}$| − |R − $\bar{\mu }$|, which is positive if participant's response is closer to the expected value than to the observed mean, and negative otherwise. The model included expected variability (high expected variability was coded as one and low expected variability as zero) and observed variability as predictors (see SI for analyses on a regression model including also predictors based on previous trials). Across participants, the regression coefficient associated with expected variability was significantly larger than zero (t(32) = 12.06, *p* < .001), indicating that, with higher expected variability, response was closer to the expected value than the observed mean. The regression coefficient associated with observed variability was also significantly positive ((t(32) = 3.58, *p* = .001), indicating that response was closer to the expected value than the observed mean when the observed variability was higher.

Next, to assess predictions of optimal inference theory more formally, and to explore any impact on choice stochasticity, we adopted a model-based approach. We assumed that participants estimated the volume of fuel in the motorbike tank under a generative model based on optimal inference principles (adapted from a Bayesian model; see Appendix). First, the generative model calculates a posterior belief about the fuel $\hat{\mu }$ that corresponds to a weighted average between the expected value $\bar{\mu }$ (either 15 or 20 L) and the observation mean $\mu_{g}$(equal to $\mu_{g}=\left(g_{1}+g_{2}\right)/2$):

(1) $$\hat{\mu }=w\mu_{g}+\left(1-w\right)\bar{\mu }$$

The parameter w reflects the weight of the observation mean $\mu_{g}$ relative to the expected value $\bar{\mu }$ and can vary between zero and one. A w>0.5 implies that the posterior belief will be closer to the observation mean than the expected value, while a w<0.5 implies the opposite (w=0.5 implies an equal distance). According to optimal inference (see Methods), the weight *w* varies as a function of the expected and observed variability. A simple way to quantify the latter is calculating the distance between gauges, namely $v_{g}=|g_{1}-g_{2}|$. This implies that the closer the numbers indicated by the gauges, the lower the observed variability. After z-scoring $v_{g}$ and calculating $v_{g}^{'}$ (which thus has mean equal to zero and SD equal to one), the weight *w* on each trial is dependent on a sigmoid function of expected and observed variability:

(2) $$w=sig\left(\bar{\sigma },\sigma_{o}^{'}\right)=\frac{1}{1+e^{\bar{v}+a_{g}v_{g}^{'}}}$$

This formulation is adapted from a Bayesian model (see Methods). The use of a sigmoid function ensures that the weight w is constrained between zero and one. The parameter $\bar{v}$ reflects an effect of the expected variability and corresponds to ${\bar{v}}_{L}$ during low expected variability trials and to ${\bar{v}}_{H}$ during high expected variability trials. This equation includes three free parameters, namely a parameter for low expected variability trials ${\bar{v}}_{L}$, a parameter for high expected variability trials ${\bar{v}}_{H}$, and a parameter $a_{g}$ which captures the effect of the z-scored observed variability $v_{g}^{'}$.

In addition, the generative model assumes stochasticity in a participant's response *R*, which is drawn from a Gaussian distribution having an average equal to the posterior belief $\hat{\mu }$ and a SD equal to $\bar{\omega }+b_{g}v_{g}^{'}$:

(3) $$R∼N\left(\hat{\mu },{\left(\bar{\omega }+b_{g}v_{g}^{'}\right)}^{2}\right)$$

The parameter $\bar{\omega }$ reflects an effect of expected variability on response stochasticity and corresponds to ${\bar{\omega }}_{L}$ during low expected variability trials and to ${\bar{\omega }}_{H}$ during high expected variability trials. This equation includes three additional free parameters. These are the parameters related to expected variability ${\bar{\omega }}_{L}$ and ${\bar{\omega }}_{H}$, plus the parameter $b_{g}$ which captures the effect of the z-scored observed variability $v_{g}^{'}$ on stochasticity. During parameter estimation, these free parameters were constrained in such a way to ensure that the overall SD of the Gaussian distribution was positive (see Appendix).

Altogether, the full model of behavioural responses included six free parameters (${\bar{v}}_{L}$, ${\bar{v}}_{H}$, $a_{g}$, ${\bar{\omega }}_{L}$, ${\bar{\omega }}_{H}$ and $b_{g}$) estimated individually from each participant's behavioural data. To assess the validity of this model, we compared it with a baseline model $Model_{base}$ in which behavioural responses were drawn from a Gaussian distribution with fixed mean and SD. As a more stringent test, the full model was also compared with four simpler models that were equivalent to the full model except for one of the following simplifications: (i) for $Model_{\bar{v}}$, ${\bar{v}}_{L}$ was constrained to be equal to ${\bar{v}}_{H}$; (ii) for $Model_{a}$, $a_{g}$ was fixed to zero; (iii) for $Model_{\omega }$, ${\bar{\omega }}_{L}$was constrained to be equal to ${\bar{\omega }}_{H}$; (iv) for $Model_{b}$, $b_{g}$ was fixed to zero.

For each model of the behavioural data, we estimated the Bayesian Information Criterion (BIC), which reports the goodness of a model in terms of an accuracy/complexity trade-off (i.e., an approximation to negative log model evidence). After summing the BIC scores across subjects for each model, we found that the full model had the lowest BIC score (i.e., highest evidence), indicating that this model outperformed simpler models in characterizing participants' behaviour (Table 1). To assess the reliability of the parameters estimated with the full model, for each participant we randomly split the trials in two sets, and estimated the parameters separately for each set. For all parameters, a significant positive correlation between the two sets was observed across participants (Fig. S1; $\;{\bar{v}}_{L}$: r(31) = .63, *p* < .001; ${\bar{v}}_{H}$: r(31) = .77, *p* < .001; $a_{g}$: r(31) = .39, *p* = .026; ${\bar{\omega }}_{H}:$ r(31) = .82, *p* < .001; ${\bar{\omega }}_{L}$: r(31) = .93, *p* < .001; $b_{g}$: r(31) = .60, *p* < .001; two-tailed alpha of .05 was used as significance criterion for behavioural analyses). Altogether, these analyses support the validity and reliability of the full model (see SI for further analyses based on simulated data).

**Table 1:** Results of the model comparison analysis. The first column reports the model considered (see main text for descriptions). The second column lists the free parameters of each model. The third column reports the negative log-likelihood of the data (summed across subjects). The fourth column reports the pseudo-$r^{2}$(Daw, 2011) which, by quantifying the improvement afforded by a model compared to a baseline model (in our case, $Model_{base}$), indicates how well the model fits the data. This quantity is bounded between zero and one, with larger values indicating a better fit. The fifth column reports the Bayesian Information Criterion (BIC; summed across subjects). The sixth column indicates for how many subjects each model showed the lowest BIC score amongst the models considered (e.g., for 22 subjects the full model had the lowest BIC).

Model	Free parameters	Neg Log-Lik	Pseudo-$r^{2}$	BIC	Number of subjects
FullModel	${\bar{v}}_{L}$, ${\bar{v}}_{H}$, $a_{g}$, ${\bar{\omega }}_{L}$, ${\bar{\omega }}_{H}$, $b_{g}$	27280	.2141	55710**	22
$Model_{\bar{v}}$	$\bar{v}$, $a_{g}$, ${\bar{\omega }}_{L}$, ${\bar{\omega }}_{H}$, $b_{g}$	27543	.2066	56090	2
$Model_{a}$	${\bar{v}}_{L}$, ${\bar{v}}_{H}$, ${\bar{\omega }}_{L}$, ${\bar{\omega }}_{H}$, $b_{g}$	27353	.2120	55765	7
$Model_{\omega }$	${\bar{v}}_{L}$, ${\bar{v}}_{H}$, $a_{g}$, $\bar{\omega }$, $b_{g}$	27498	.2078	56001	1
$Model_{b}$	${\bar{v}}_{L}$, ${\bar{v}}_{H}$, $a_{g}$, ${\bar{\omega }}_{L}$, ${\bar{\omega }}_{H}$	27678	.2027	56361	0
$Model_{base}$	m,SD	34713	0	69827	0

**Fig. 2:**
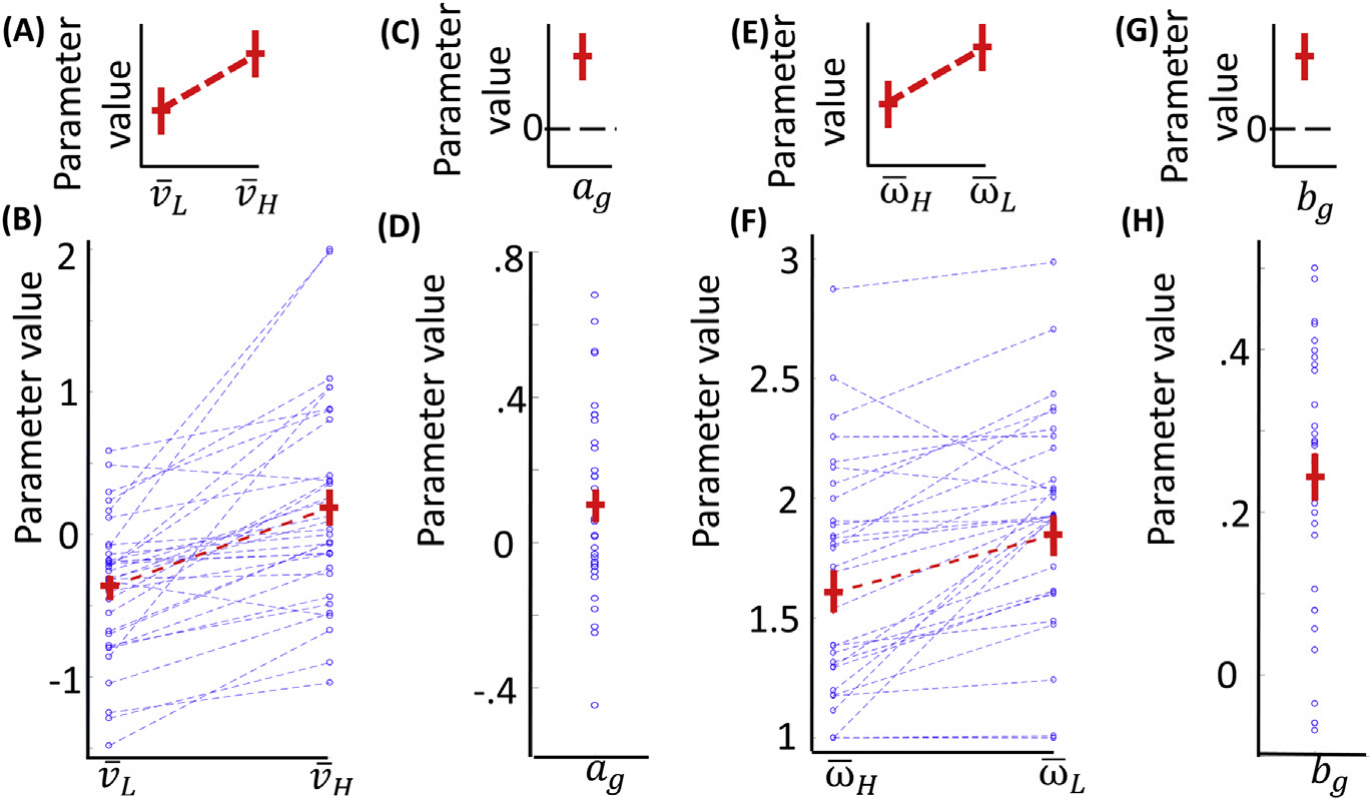
Effects predicted by the optimal inference hypothesis and their test. **A**: Prediction one, whereby the value of the parameter ${\bar{v}}_{H}$ was expected to be larger than the value of ${\bar{v}}_{L}$ (red horizontal lines indicates means; red vertical lines indicate standard errors). **B:** Data for prediction one, where blue dots indicate parameter values for individual participants (t(32) = 5.81, *p* < .001). **C**: Prediction two, whereby the value of the parameter $a_{g}$ was expected to be larger than zero. **D**: Data for prediction two (t(32) = 2.18, *p* = .037). **E**: Prediction three, whereby the value of the parameter ${\bar{\omega }}_{L}$ was expected to be larger than the value of ${\bar{\omega }}_{H}$. **F**: Data for prediction three (t(32) = 4.73, *p* < .001). **G**: Prediction four, whereby the value of the parameter $b_{g}$ was expected to be larger than zero. **H**: Data for prediction four (t(32) = 8.26, *p* < .001).

We then used the full model to test the predictions derived from the optimal inference hypothesis outlined above. First (*Prediction one*), the value of ${\bar{v}}_{H}$ will be larger than the value of ${\bar{v}}_{L}$ (Fig. 2A). This implies that the weight w was larger with ${\bar{v}}_{L}$ than with ${\bar{v}}_{H}$, entailing that the posterior belief was closer to the observation mean than the expected value in low compared to high expected variability trials. This prediction was supported by our results, where we observed a larger value for ${\bar{v}}_{H}$ compared to ${\bar{v}}_{L}$ across participants (Fig. 2B; t(32) = 5.81, *p* < .001). A second prediction (*Prediction two*) was that $a_{g}$ would be larger than zero (Fig. 2C). This indicates the weight w decreased with higher z-scored observed variability $v_{g}^{'}$ – implying that the posterior belief was closer to the expected value than to the observation mean with higher z-scored observed variability $v_{g}^{'}$. Our results were consistent with this prediction, as $a_{g}$ was significantly larger than zero across participants (Fig. 2D; t(32) = 2.18, *p* = .037). Third (*Prediction three*), we predicted that the value of ${\bar{\omega }}_{H}$ would be larger than the value of ${\bar{\omega }}_{L}$, implying a higher stochasticity during high compared to low expected variability trials (Fig. 2E). Data supported this, showing a larger value for ${\bar{\omega }}_{H}$compared to ${\bar{\omega }}_{L}$ (Fig. 2F; t(32) = 4.73, *p* < .001). Finally (*Prediction four*), a value larger than zero was predicted for $b_{g}$, implying a higher stochasticity for higher z-scored observed variability $v_{g}^{'}$ (Fig. 2G). This accorded with our observation of a value of $b_{g}$ that was significantly larger than zero across participants (Fig. 2F; t(32) = 8.26, *p* < .001). Given our focus on inference and not on learning, we predicted the effects tested here to remain stable along the task, and our analyses comparing the first versus second half of the task confirmed this prediction (see SI).

In sum, our behavioural analyses were consistent with predictions derived from optimal inference, highlighting a dual role for both expected and observed variability. The first role implies the expected value is relied upon more under higher expected and observed variability. The second role implies that stochasticity increases with both expected and observed variability. Intuitively, if the observed and expected variability did not impact on choice stochasticity, a participant's estimate would correspond to the posterior belief plus some error, but the error term would be fixed. In other words, the brain would first rely on expected value and gauges to infer the posterior belief, corresponding to a single point estimate, and next it would sample from a distribution with a fixed variance centred on the posterior estimate. On the contrary, our results support the notion that observed and expected variability affect choice stochasticity, in other words that the brain samples from a distribution tuned to the current expected and observed variability.

### 3.2 Neuroimaging

We characterised the neural processes underlying the weighting of prior expectations and observations during inference. Specifically, we analysed brain activity in our regions of interest (ROIs) – comprising the hippocampus and SPC – as a function of expected and observed variability, and in relation to expression of a PE (see Methods). We fitted a GLM having, at the time when the two numbers indicated by the gauges $g_{1}$ and $g_{2}$ appeared, a stick function regressor for high expected variability trials and another for low expected variability trials. Each was modulated by observed variability $v_{g}$ equal to $v_{g}=|g_{1}-g_{2}|$ (i.e., the distance between the numbers indicated by the gauges). A second parametric modulator was a PE equal to $PE=|\bar{\mu }$ − $\mu_{g}|$, namely, the distance between the expected value $\bar{\mu }$ (either 15 or 20 L) and the observation mean $\mu_{g}$(noting that $\mu_{g}=\left(g_{1}+g_{2}\right)/2$).

**Fig. 3:**
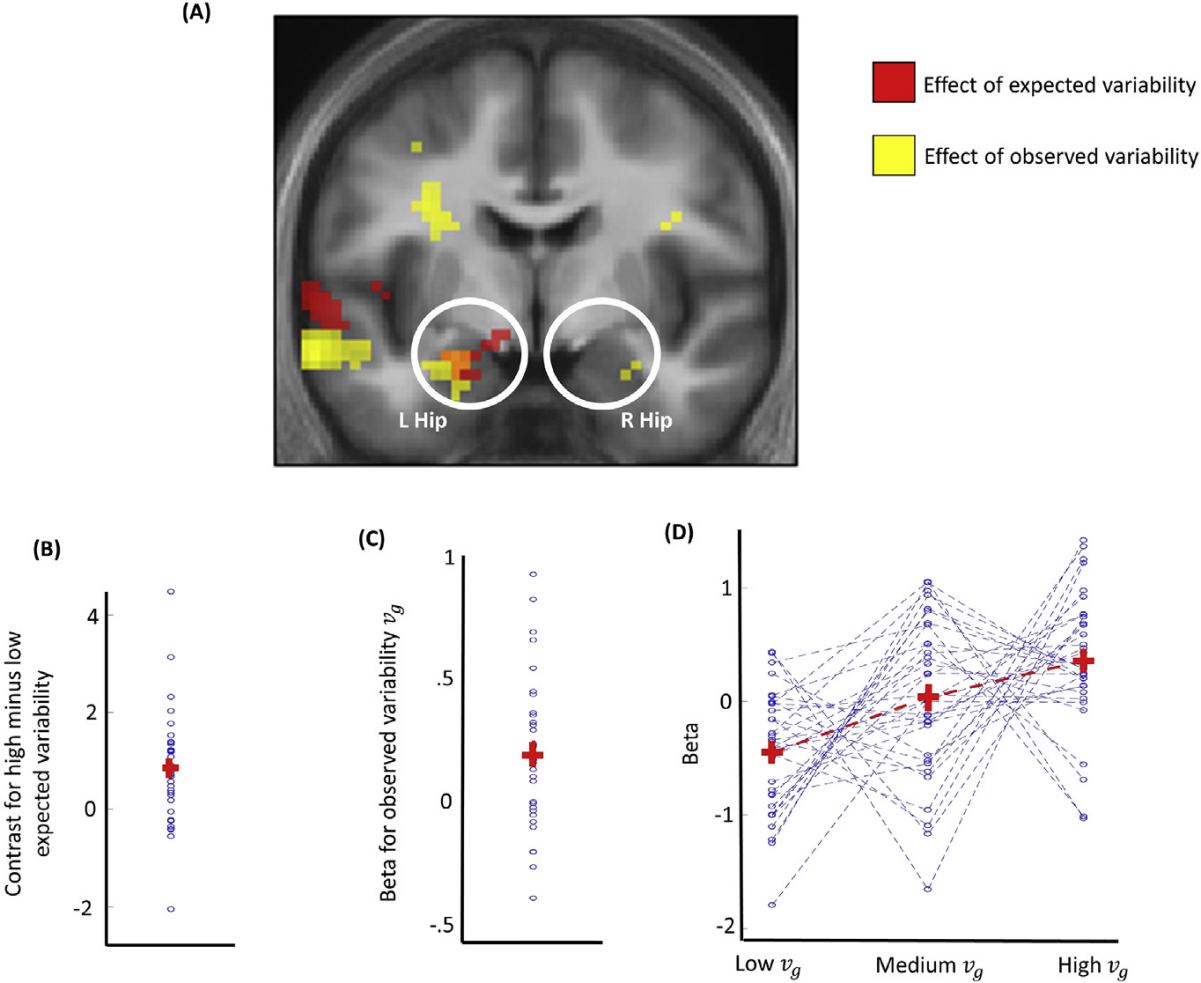
fMRI results about the effect of expected and observed variability in the hippocampus. **A**: Voxels activated at *p* < .001 uncorrected (these are shown for display purposes only) are displayed in red for the effect of expected variability and in yellow for the effect of observed variability. The brain image corresponds to the mean structural image of participants. **B**: Value of the high versus low expected variability contrast in the peak activation voxel of left hippocampus (L Hip; −21, −7, −20; Z = 3.58, *p* = .009 SVC; Montreal Neurological Institute coordinates were used)). The horizontal red line indicates the average across participants, the vertical red line indicates the standard error, and the blue dots indicate values for individual participants. **C**: Value of the GLM beta parameter relative to the observed variability in the peak activation voxel of left hippocampus (−21, −10, −20; Z = 4.10, *p* = .002 SVC). **D**: Activation in left hippocampus (−21, −10, −20) for different levels of observed variability. These were obtained based on a GLM where observed variability was ordered in three bins of equal numericity, and where each bin was associated with a stick function regressor. This GLM was estimated for display purposes only, and was not used for statistical testing.

**Fig. 4:**
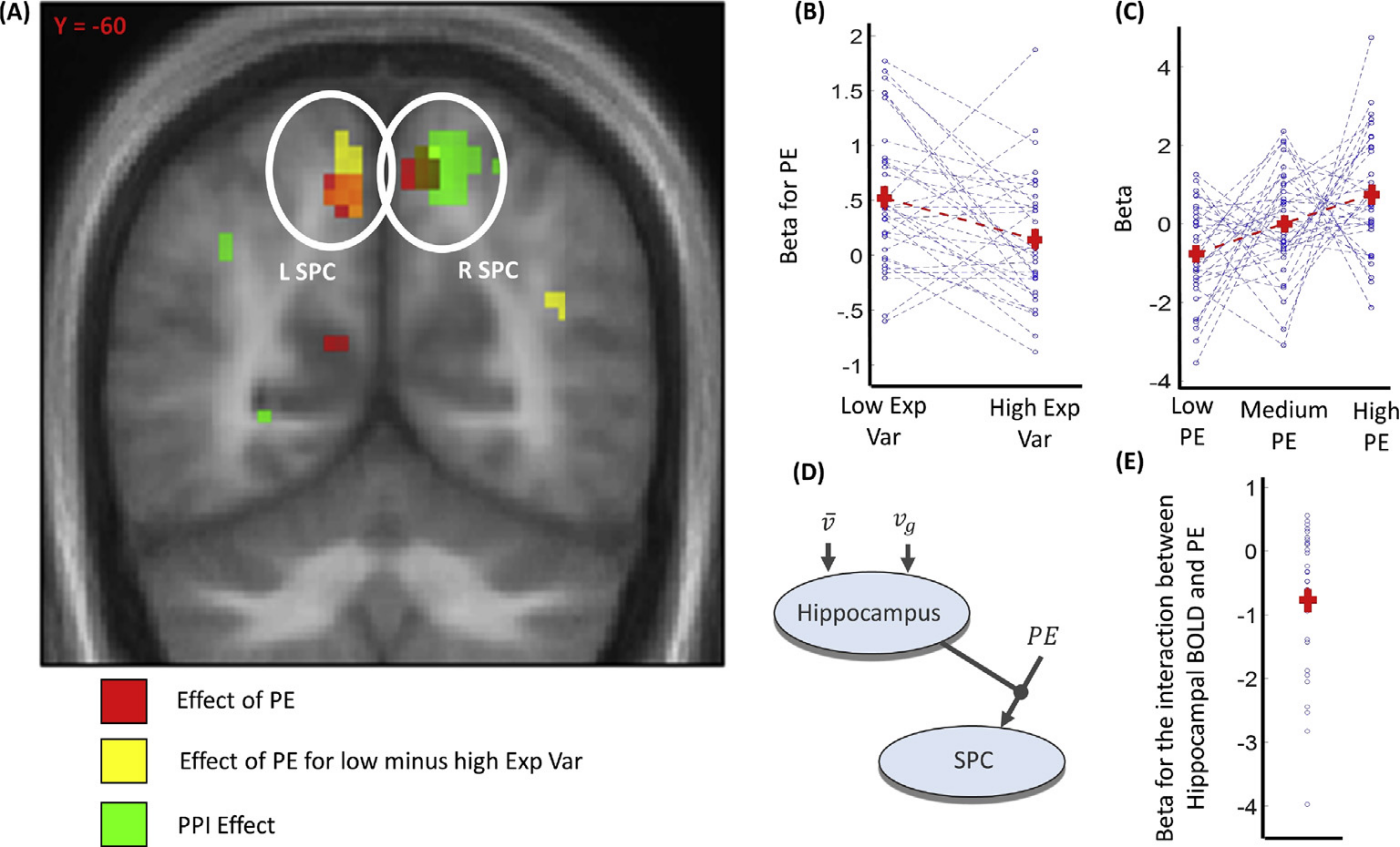
fMRI results about the effects emerged in superior parietal cortex (SPC). **A**: Voxels activated at *p* < .001 uncorrected (shown for display purposes only) are displayed in red for the effect of prediction error (PE; calculated as the distance between the expected value and the observation mean, namely $PE=|\bar{\mu }$ - $\mu_{g}|$), in yellow for the contrast comparing PE response during low compared to high expected variability, and in green for the PPI analysis having left hippocampus as seed region and having PE as psychological condition. The brain image corresponds to the mean structural image of participants. **B**: fMRI results for the beta parameter associated with PE when comparing low versus high expected variability trials in the peak activation voxel of SPC (9, −55, 55; Z = 3.57, *p* = .010). The beta parameters are plotted separately for low expected variability (Low Exp Var) and high expected variability (High Exp Var) trials. Horizontal red lines indicate averages across participants, vertical red lines indicate the standard error, and blue dots indicate values for individual participants. **C**: Activation in the peak activation voxel of left SPC (−9, −64, 49; Z = 3.25, *p* = .025 SVC) for different levels of PE. These were obtained based on a GLM where PE was ordered in three bins of equal numericity, and where each bin was associated with a stick function regressor. This GLM was estimated for display purposes only, and was not used for statistical testing. **D**: Scheme of the hypothetical neural circuit involved in our task, where (i) the hippocampus implements the weight of the expected value as its activity increases with expected variability $\bar{v}$ and observed variability $v_{g}$, (ii) the SPC response reflects PE, (iii) the hippocampus modulates the responsivity of SPC to PE, as stronger hippocampal response attenuates the responsivity. The latter element of the circuit was examined with the PPI analysis. **E**: PPI effect in the peak activation voxel of right SPC (15, −61, 55; Z = 3.54, *p* = .011 SVC).

When assessing the influence of expected variability, we observed an increased response for high compared to low expected variability in left hippocampus (Fig. 3; −21, −7, −20; Z = 3.58, *p* = .009 SVC; Montreal Neurological Institute coordinates were used) but not right hippocampus nor SPC (*p* > .05 SVC). Note that this contrast is not biased by any difference in observed variability between the two conditions, as observed variability $v_{g}$ was not rescaled to the mean within the GLM (see Methods). When we examined the GLM beta parameter associated with the observed variability $v_{g}$ we found this was significantly greater than zero in bilateral hippocampi (Fig. 3; left: −21, −10, −20; Z = 4.10, *p* = .002 SVC; right: 21, −13, −17; Z = 3.51, *p* = .011 SVC) but not in SPC. Finally, we found that the beta parameter associated with a PE was significantly positive in bilateral SPC (Fig. 4; left: −9, −64, 49; Z = 3.25, *p* = .025 SVC; right: 9, −61, 52; Z = 3.23, *p* = .026) but not in the hippocampus.

We used our computational model of behaviour to probe these results further. With a simple algebraic transformation, we can rewrite equation (1) as:

(4) $$\hat{\mu }=\bar{\mu }+w\left(\mu_{g}-\bar{\mu }\right)=\bar{\mu }+\frac{1}{1+e^{\bar{v}+a_{g}v_{g}^{'}}}\left(\mu_{g}-\bar{\mu }\right)$$

This shows that the posterior belief is equal to the expected value plus the difference between the observation mean $\mu_{g}$ and the expected value $\bar{\mu }$ multiplied by the weight w. Note that this equation resembles a standard Rescorla-Wagner rule (Friston, 2005, Mathys et al., 2011, Rigoli, Friston et al., 2016). From equation (2), we know that w corresponds to the relative weight of the observation mean, as it decreases with the expected variability $\bar{v}$and with the z-scored observed variability $v_{g}^{'}$. Equation four allows one to define a precision-weighted prediction error $PE_{w}$ as:

(5) $$PE_{w}\overset{def}{=}\left|w\left(\mu_{g}-\bar{\mu }\right)\right|=\left|\frac{1}{1+e^{\bar{v}+a_{g}v_{g}^{'}}}\left(\mu_{g}-\bar{\mu }\right)\right|$$

This is the precision-weighted distance between the observation mean and the expected value, in other words a quantification of how much a belief should change after new observations. Crucially, this is formally analogous to the construct of surprise, as it quantifies how surprising observations are (Friston, 2005). Note that the surprise depends upon the quality of the observations. In other words, unreliable observations are less surprising than reliable observations.

Considering equations (4), (5)), the fMRI results raise the following questions: (i) do responses in SPC reflect a PE or a precision-weighted PE signal? (ii) Do left hippocampus responses reflect the weight of the expected value, which formally corresponds to the opposite of the weight w or to (1−w)? This possibility is consistent with the increased response with expected and observed variability found in hippocampus in the previous analysis. (iii) Does activity in hippocampus modulate the responsivity (or gain) in SPC to PE?

To answer the first question, we reasoned that a precision-weighted PE would predict a stronger relationship between PE and SPC activity under low compared to high expected variability. We tested this prediction by comparing the beta parameter for PE for low minus high expected variability trials and consistent with our hypothesis we found a significant difference in bilateral SPC (Fig. 4; left: −9, −58, 52; Z = 3.17, *p* = .030 SVC; right: 9, −55, 55; Z = 3.57, *p* = .010).

To address the second question, we fitted a second GLM equal to the previous one except that a single stick function regressor was included when $g_{1}$ and $g_{2}$ appeared, in this case modulated by the expression $\bar{v}+a_{g}v_{g}^{'}$, (i.e., the weight 1−w without the sigmoid transformation) and by RTs as a nuisance parametric modulator. For calculating $\bar{v}+a_{g}v_{g}^{'}$, we used the computational model of behaviour to estimate the parameters ${\bar{v}}_{L}$, ${\bar{v}}_{H}$, and $a_{g}$. Following Wilson and Niv (2015), for these parameters we used the same values for all subjects, corresponding to the mean parameter scores (to ascertain that our results did not depend on this approach, we also performed the same analysis except that individual parameter scores were used for ${\bar{v}}_{L}$, ${\bar{v}}_{H}$, and $a_{g}$; similar results were obtained (not shown)). The GLM beta parameter associated with $\bar{v}+a_{g}v_{g}^{'}$ was significantly positive in left hippocampus (−21, −7, −17; Z = 4.56, *p* < .001 SVC). In other words, hippocampal responses were greater when observations had higher expected and observed variability, consistent with the notion that the hippocampus encodes the relative weight of the expected over the observed value.

We investigated the connectivity between hippocampus and SPC, examining whether the hippocampus modulates the responsivity – or gain – in SPC to a PE (Fig. 4). To test this, we performed a psychophysiological interaction (PPI) analysis (based on the first GLM), using the left hippocampus as (physiological) seed region (specifically, the voxel with coordinates −21, −7, −17, which showed the peak activation in the analysis based on the second GLM for the expression $\bar{v}+a_{g}v_{g}^{'}$) and the PE as the experimental (psychological) condition. A significant negative interaction (i.e., PPI) parameter was observed in right SPC (Fig. 4; 15, −61, 55; Z = 3.54, *p* = .011 SVC; all voxels showed *p* > .05 SVC in left SPC), indicating a stronger relationship between SPC response and PE when activity in the left hippocampus was lower. Although alternative interpretations cannot be excluded, this finding is in line with our hypothesis that an hippocampal encoding of the weight of expectation modulates a responsivity of SPC to PE. In summary, these results suggest that hippocampal activation depends on the expected and observed reliability of evidence, and modulates the sensitivity of SPC to PEs.

We examined the time of feedback by exploring neural activity related with outcome PE (corresponding to the distance between the feedback number and the number chosen by the participant). A positive relationship was evident between outcome PE and activity in bilateral SPC (left: −9, −58, 49; Z = 4.42, *p* < .001 SVC; right: 9, −58, 52; Z = 5.52, *p* < .001 SVC). This indicates that SPC processes information related with PE also at feedback. Interestingly, adopting whole-brain correction, an inverse relationship emerged between outcome PE and activity in ventral striatum (left: −12, 11, −2; Z = 6.74, *p* < .001 whole-brain corrected; right: 9, 11, −5; Z = 6.69, *p* < .001 whole-brain corrected). The latter region is important for reward processing, as for instance substantial evidence shows that activity in this region reflects how much a reward is better than expected (e.g., Glimcher, 2011). In our task, it is reasonable to assume that participants were rewarded more when their response was closer to the number revealed, which is consistent with the observation of an inverse correlation between ventral striatum and outcome PE.

Interestingly, recent studies have supported the possibility that the amygdala also contributes to aspects of novelty processing (Balderston et al., 2011, Schwartz et al., 2003). Hence, for exploratory purposes, we asked whether the effects of observed and expected variability could be found in the amygdala too. We defined amygdala using the anatomical mask available in the MarsBar AAL archive (Tzourio-Mazoyer et al., 2002). An effect associated with expected variability emerged in left, but not right, amygdala (−21, −7, −17; T = 3.76, *p* < .001 uncorrected; given the exploratory nature of this analysis, *p* < .001 uncorrected was used a threshold), though no effect associated with observed variability was evident (these results were confirmed also when the analyses were re-run using a 4 mm smoothing kernel (data not shown)). This hints to the possibility that the amygdala partially contributes to processing expectations about variability. Note that our hippocampal ROI (see methods) and the amygdala mask used here are mutually exclusive, meaning that each voxel either belongs to one or to the other region. This implies that it is unlikely that the effects observed in the hippocampus are primarily caused by neuronal activity occurring in the amygdala. However, because fMRI is an indirect measure of neural activity and has limited spatial resolution, our study is unable to fully rule out the possibility that neurons in posterior amygdala also contribute partially to the effects observed in hippocampus.

## 4 Discussion

Optimal weighting of prior expectations against novel sensory evidence is crucial for efficient inference. How this optimal weighting is realized in the brain has remained elusive. Our findings show enhanced hippocampal responses with high expected and observed variability, conditions in which a participant's estimates rely more on prior expectation than novel observations. In addition, though we emphasize that PPI analyses do not demonstrate directionality of effect, our PPI findings are consistent with the possibility that enhanced hippocampal activation attenuates SPC responses to PEs, in other words that the hippocampus implements a form of optimal weighting to regulate the response gain of regions processing PEs (i.e., SPC).

At the behavioural level, our study aimed to reveal how the reliability of observations is established based on expected and observed variability. This aspect has been neglected by previous research which mostly focused on the role of the expected value and its uncertainty. The latter captures how much a *hidden variable* (e.g., the fuel amount) is expected to vary, while here we analysed how much *observations* (e.g., the number reported by the gauges) are expected to vary. Our findings fit the notion that agents evaluate observations as more reliable when the observed and expected variability are lower. We found that this reliability of observations was influential at two distinct levels. Firstly, it determined how much participants' estimates were closer to expectations compared to observations. Secondly, less reliable observations were associated with more stochastic responses. These findings support optimal inference principles, and extend these to conditions in which expectations about the variability of observations are manipulated. In addition, our results imply that response stochasticity, which has been neglected in previous studies, is an important aspect of optimal inference.

Several theoretical proposals have been offered to explain how the brain performs inference at a neural circuit level (Knill and Pouget, 2004, Ma and Jazayeri, 2014, Friston, 2005, Friston, 2010, Jazayeri, 2008, Ma et al., 2006, O'Reilly, Schüffelgen, et al., 2013, Pouget et al., 2013, Rao and Ballard, 1999, Vilares and Kording, 2011, Vilares et al., 2012). These theories debate on which brain regions encode expectations about variability, and on whether the same regions also encode variability observed in the data. Our findings shed light on this issue by showing enhanced hippocampal activity both with increasing expected *and* observed variability of upcoming evidence. These empirical findings support proposals wherein a weighting of expectations relative to observations is realized within the same brain structures. Another important question has been whether regions involved in weighting expectations also reflect a precision-weighted PE (i.e., surprise) signal. Our results support an anatomical segregation of these signals, as weighting was implemented in the hippocampus whereas precision-weighted PEs were signalled in SPC.

The previous literature has also left open the question of whether areas involved in optimal weighting during inference are modality-specific or cross-modal, in other words whether similar brain regions are recruited across different sensory modalities. While most previous neuroimaging experiments focused on sensory and perceptual tasks, our study asked subjects to make inference about an abstract variable. Hence, our results might be explained by the fact that the hippocampus is engaged only during such abstract inference processes. However, our results are also compatible with the idea that the hippocampus contributes to optimal weighting in a modality-independent fashion. Existing evidence favours the latter explanation as previous neuroimaging studies employing sensory tasks have shown that hippocampal responses increase with sensory entropy, a measure analogous to observed variability in our design (Harrison et al., 2006, Strange et al., 2005, Tobia et al., 2012).

Our findings indicate that a core function of the hippocampus is to establish whether an agent should rely more on internal representations or external upcoming information. In contexts such as our inference task, this implies optimising the weight attributed to expectations over novel evidence. A similar mechanism can be proposed to explain the critical role of the hippocampus in memory recollection, a process wherein agents naturally rely more on internal memory representations than new external information (Schacter, Alpert, Savage, Rauch, & Albert, 1996). Likewise, planning requires a consideration of internal representations about possible future states. There is evidence that hippocampal activation increases with the number and complexity of the representations activated during planning (Johnson et al., 2007, Kaplan et al., 2017, Miller et al., 2017, Pezzulo et al., 2014), consistent with a view that hippocampal activation emphasizes internal representations, in this case in the form of possible future states. Analogous interpretations can be proposed for mind-wandering, imagination, and self-projection (Buckner and Carroll, 2007, Rigoli, Ewbank et al., 2016, Smallwood, 2013), in which the hippocampus plays an important role and where internal representations assume prominence. Moreover, an influential body of work indicates the hippocampus supports a form of inference termed pattern completion (Bakker et al., 2008, Hasselmo and Wyble, 1997, McNaughton and Nadel, 1990, Neunuebel and Knierim, 2014, Rolls and Treves, 1998), where partial cues are sufficient to activate a full object representation. Enhanced hippocampal activity is reported when an internal representation (e.g., of an object) is evoked by partial cues (Bakker et al., 2008, Neunuebel and Knierim, 2014). Pattern completion is analogous to inference under uncertainty in as much as both invoke an integration of prior information and sensory evidence. Our findings rise the possibility that hippocampus implements a weighting of prior expectations and novel evidence which may also be critical for pattern completion.

It has been proposed that the hippocampus embodies a comparator mechanism (Gray and McNaughton, 2003, Kumaran and Maguire, 2009, Lisman and Otmakhova, 2001, Vinogradova, 2001). This implies a sensitivity of this region to surprising stimuli, a possibility supported by substantial evidence (Kumaran & Maguire, 2009). However, at least for simple non-associative stimuli (associative stimuli may engage different processes; see Kumaran & Maguire, 2009), previous findings suggest that the hippocampus responds to surprising events only early in a task, when learning is engaged (Strange & Dolan, 2001). For example, it has been observed that odd-ball stimuli activate this region only during early trials (Strange & Dolan, 2001). Consistent with this evidence, we found no hippocampal response to PE in our data. This observation can be explained by the fact that learning was irrelevant in our task, given that participants had already played the task extensively before scanning.

Our data indicate that the relative weight of expectations over novel evidence is encoded in the anterior hippocampus. The specific involvement of this portion is consistent with prior observations that the anterior hippocampus is widely engaged during novelty, surprise, and uncertainty processing (e.g., Harrison et al., 2006, Kumaran and Maguire, 2009, Strange et al., 2005, Tobia et al., 2012).

Our PPI analysis supports the notion the hippocampus plays a role in modulating the responsivity – or gain – of SPC to PE. This is consistent with the notion that neural units involved in weighting prior expectations are segregated from units encoding PEs, and where the former modulate the responsivity or postsynaptic gain of the latter (Friston, 2005, Friston, 2010, Rao and Ballard, 1999).

A recent theoretical model proposes that the SPC plays a critical role in encoding surprise, corresponding to a precision-weighted PE in our study (O'Reilly, Schüffelgen, et al., 2013). Though previous reports have demonstrated a relationship between SPC activity and surprise (Strange et al., 2005, O'Reilly, Schüffelgen, et al., 2013, O'Reilly, Jbabdi, et al., 2013), they were not in a position to dissociate between PE and precision-weighted PE. Our study addresses this issue, showing an enhanced SPC responsivity to PE during low compared to high expected variability, supporting the hypothesis that SPC activity signals a precision-weighted PE.

Previous evidence has also linked activity in SPC to orienting overt and covert attention, especially in relation to space, but also with dimensions such as time (Coull and Nobre, 1998, Leon and Shadlen, 2003, Mars et al., 2011, Rushworth et al., 2001, Wojciulik and Kanwisher, 1999). The SPC is also implicated in processing numbers (Dehaene et al., 2003, Pinel et al., 2001). This finding has been interpreted as the brain representing numerical quantities based on a “numerical line”, which reflects an abstraction developed from spatial representations (Dehane et al., 2003). This idea has received empirical support in psychological studies (Dehaene, Bossini, & Giraux, 1993) and has inspired the idea that SPC may be involved in orienting attention not only within space but also within an abstract “numerical line” (Dehane et al., 2003). It is of interest that, when asked to locate the middle of a line segment, patients with right parietal lesions and unilateral neglect tend to indicate a location further to the right, consistent with their failure to attend to the left side of space. In their study, Zorzi, Priftis, and Umiltà (2002) adopted a numerical bisection task where such patients had to find the middle of two orally presented numbers. Patients tended to report a number larger than the correct answer, in other words, on the right of the centre of the “number line” (e.g., if the two test numbers were 11 and 19, patients may answer 17). This effect occurred putatively because of a failure to attend to the left side of the number line – analogous to the failure seen in the spatial task. This finding supports the notion that the parietal cortex – and possibly the SPC–is important in guiding attentional mechanisms underlying number processing. Within this view, our finding of precision-weighted PE in SPC can be interpreted as indicating how much within an abstract “numerical line” attention should be shifted from prior expectations.

A large body of evidence has shown that activity in the striatum of the basal ganglia reflects a reward PE, namely how much a reward is better than expected (e.g., Glimcher, 2011). An important question is whether this motivational quantity is analogous to the notion of magnitude PE as conceived in our study. Crucially, at feedback time, larger distance between the participants' response and the feedback implies larger magnitude PE but (as it indexes poor performance and hence less reward) also smaller reward PE, thus dissociating the two quantities. Activity in SPC and in striatum was positively and negatively related with the distance between response and feedback, respectively. This indicates that reward PE is not related with the magnitude PE signalled in SPC, and that the latter is implicated during inference of magnitudes, but unrelated with the motivational consequences elicited by this inference.

Finally, we acknowledge limitations of our study. At the behavioural level, our focus was on the actual strategies adopted by participants during inference. A question that remains open is whether participants are aware of these strategies, and more generally it remains poorly understood what participants believe about how they approach inference problems. At the neural level, the limited spatial resolution of fMRI limits our ability to explore whether distinct hippocampal regions are engaged by expectations about variability and by variability observed in data. A segregation might be revealed by more fine-grained methodology in the future. In addition, due to limited temporal resolution of fMRI, our results leave open the question of how a hippocampal response to upcoming evidence evolves in time within a trial. Another shortcoming is that our PPI analyses cannot demonstrate directionalities. Thus, although our PPI results fit with a predictive coding formulation in which the hippocampus regulates the gain response in SPC, alternative explanations cannot be fully ruled out.

In summary, our findings help clarifying the behavioural and neural mechanisms underlying inference under uncertainty. At the behavioural level, we show that the expected and observed variability establish the reliability of observations, determining the attractiveness of expectations over observations and the stochasticity of responses. At the neural level, our findings highlight that the hippocampus (integrating both expected and observed variability of upcoming information) encodes the weight of prior expectations and modulates responses in SPC to PE (resulting in the expression of a precision-weighted PE). Together with empirical evidence from domains such as memory, planning, and self-projection, our results support a view that a critical role of hippocampus is to reflect the relevance of acquired internal representations compared to upcoming novel evidence.

## Data availability statement

The datasets generated during and/or analysed during the current study are available from the corresponding author on reasonable request.

## Appendix

Participants' responses were modelled using a generative model inspired by Bayesian inference. Participants' estimates can be characterized by a Bayesian model in which a posterior gauge uncertainty and a posterior value of the true fuel amount are inferred in two separate steps. During the first step, the model assumes that the numbers reported by the two gauges $g_{1}$ and $g_{2}$are sampled from a Gaussian distribution with mean $\mu_{g}=\left(g_{1}+g_{2}\right)/2$) and unknown variance ${\bar{\sigma }}_{g}^{2}$:

(6) $$g_{x}∼N\left(\mu_{g},{\bar{\sigma }}_{g}^{2}\right)$$

The gauge variance ${\bar{\sigma }}_{g}^{2}$ is assumed to be sampled from an Inverse Gamma (IG) distribution with known hyperparameters $\alpha_{g}$ and $\beta_{g}$:

(7) $${\bar{\sigma }}_{g}^{2}∼IG\left(\alpha_{g},\beta_{g}\right)$$

Using Bayesian belief updating (and setting $\alpha_{g}$ equal to zero for simplicity), this model can be inverted to estimate the posterior gauge variance ${\hat{\sigma }}_{g}^{2}$ (the hat symbol indicates estimated parameters):

(8) $${\hat{\sigma }}_{g}^{2}=\beta_{g}+\frac{\sum {\left(g_{x}-\mu_{g}\right)}^{2}}{2}=\beta_{g}+VAR_{g}$$

$VAR_{g}$ is the observed variance of the gauge distribution, and $\beta_{g}$ can be interpreted as an expected variance.

During the second step, the Bayesian model assumes that the number reporting the true amount of fuel μ is sampled from a Gaussian distribution with known mean $\bar{\mu }$ (corresponding to the expected value of 15 or 20) and variance ${\bar{\sigma }}^{2}$ (for simplicity, this is set to one):

(9) $$\mu ∼N\left(\bar{\mu },{\bar{\sigma }}^{2}\right)$$

Also at the second step, the model assumes that the two gauges $g_{1}$ and $g_{2}$are sampled from a Gaussian distribution with mean μ and variance ${\hat{\sigma }}_{g}^{2}$, i.e., the posterior gauge uncertainty estimated before:

(10) $$g_{x}∼N\left(\mu ,{\hat{\sigma }}_{g}^{2}\right)$$

Using Bayesian belief updating, a posterior estimate of the fuel amount $\hat{\mu }$ is obtained as follows:

(11) $$\hat{\mu }=\frac{1}{{\hat{\sigma }}_{g}^{2}+1}\mu_{g}+\frac{{\hat{\sigma }}_{g}^{2}}{{\hat{\sigma }}_{g}^{2}+1}\bar{\mu }=\frac{1}{\beta_{g}+VAR_{g}+1}\mu_{g}+\left(1-\frac{1}{\beta_{g}+VAR_{g}+1}\right)\bar{\mu }$$

Note the similarity with the model described by equations (1), (2)). In particular, the ratio $\frac{1}{\beta_{g}+VAR_{g}+1}$ is analogous to the weight *w* because like the latter (i) it is a number larger than zero and smaller than one, (ii) it decreases with the expected variability (captured by $\beta_{g}$ in equation 11), (iii) it decreases with the observed variability (captured by $VAR_{g}$ in equation 11), (iv) it is multiplied by $\mu_{g}$, (v) one minus this ratio is multiplied by $\bar{\mu }$.

From this Bayesian model, one can derive the four empirical predictions we tested. We reasoned that the most conservative way to test these predictions empirically would be to estimate free parameters from participants' behaviour and performing statistical testing on these. However, the Bayesian model described by equation (11) has no free parameters. Hence, we adopted a model (described by equations (1), (2))) that retains all the key phenomenological properties but allows one to estimate free parameters – and thus to test the predictions empirically. Note that, to quantify the observed variability, the original Bayesian model uses variance ($VAR_{g}=\frac{\sum {\left(g_{x}-\mu_{g}\right)}^{2}}{2}$), while the phenomenological model uses distance ($v_{g}=|g_{1}$ - $g_{2}|$). The reason is that, in our task, the distribution of gauge distance across trials was less skewed than the distribution of gauge variance, meaning that parameter estimates were likely to be more robust if gauge distance and not gauge variance was used (Fig. S2). However, we emphasize that the four empirical predictions examined here can be derived irrespective of whether variance or distance was considered.

Note that Bayesian belief updating can be formalized also using precision, which is the inverse of variance:

(12) $$\hat{\mu }=\frac{\pi_{g}}{\pi_{g}+1}\mu_{g}+\frac{1}{\pi_{g}+1}\bar{\mu }=\frac{\pi_{g}}{\pi_{g}+1}{\text{μ}}_{g}+\left(1-\frac{\pi_{g}}{\pi_{g}+1}\right)\bar{\text{μ}}$$

Where the gauge precision is $\pi_{g}=1/\left(\beta_{g}+VAR_{g}\right)$. This formulation illustrates the link between precision and uncertainty (i.e., variance). Equations (11), (12)) allow one to define a precision-weighted PE based on Bayesian belief updating (from which we adapted equation (5)):

(13) $$PE_{w}\overset{def}{=}\left|\frac{\pi_{g}}{\pi_{g}+1}\left({\text{μ}}_{g}-\bar{\text{μ}}\right)\right|=\left|\frac{1}{\beta_{g}+VAR_{g}+1}\left({\text{μ}}_{g}-\bar{\text{μ}}\right)\right|$$

For the modelling analyses, free parameters were estimated from each participant's behaviour using *fminsearchbnd* function in Matlab. The parameters related to the effect of expected variability over stochasticity ${\bar{\omega }}_{L}$ and ${\bar{\omega }}_{H}$ were constrained to be larger than one, so to guarantee that the SD of the Gaussian distribution generating the response was positive. Other parameters were not bounded. During parameter estimation, the starting value was set to zero for all parameters except for ${\bar{\omega }}_{L}$ and ${\bar{\omega }}_{H}$ for which the starting value was set to one.
