# Supplementary material for: The role of the hippocampus in weighting expectations during inference under uncertainty
Source: Cortex. 2019 Jun;115:1–14. doi: 10.1016/j.cortex.2019.01.005 (PMC6533111; doi:10.1016/j.cortex.2019.01.005)
Supplement: Multimedia component 1 [file mmc1.docx]

**SUPPLEMENTARY INFORMATION**

**Analyses of reaction time data**

We analysed the effects of the factors manipulated in our study on reaction time (RT), corresponding to the interval from the presentation of the numbers indicated by the gauges to the first button press. We considered three alternative hypotheses. A first possibility is that RTs may reflect the overall reliability of observations, which in our task implies slower RTs with higher expected and observed variability, but no effect of PE. A second possibility is that RTs reflect surprise, in our task corresponding to a weighted prediction error. This predicts slower RTs with lower expected and observed variability, and with higher PE. A third possibility is that RTs are affected by calculations based on the information carried by the gauges. In other words, while the expected variability is known *a priori*, the observation variability and the PE can be calculated only when the gauge numbers appear, and RTs may be influenced by this calculation. This predicts slower RTs with higher observation variability and with higher PE, but no effect of expected variability.

We fitted a GLM to the log-transformed RTs having (i) one binary regressor indicating high or low expected variability trials, (ii) one regressor reporting a PE equal to $PE=|\bar{\mu}$ - $\mu_{g}|$, (iii) one regressor reporting the distance across gauges $v_{g}=|g_{1}$ - $g_{2}|$ (reflecting the observation variability). When testing the beta parameters across subjects, we observed (i) no difference between high and low expected variability trials (t(32) = -0.94, p = 0.353), (ii) slower RTs with larger PE (t(32) = 5.50, p < 0.001), and (iii) slower RTs with larger gauge distance (t(32) = 5.09, p < 0.001).

Showing slower RTs with higher observation variability and higher PE, these results support the possibility that in our task RTs were influenced by the calculation elicited when new observations are presented.

**Analysis of influence of previous trials**

We asked whether participant’s responses were affected by conditions at previous trials. Similar to the main text, we adopted a multiple regression model having y = |R - $\mu_{g}$| - |R - $\bar{\mu}$| as dependent variable (where $\bar{\mu}$ corresponds to the expected value (either 15 or 20 litres) and $\mu_{g}$(equal to $\mu_{g}={(g}_{1}$ + $g_{2})/2$) to the observed mean of the gauges). Remember that this variable is positive if participant’s response is closer to the expected value than to the observed mean, and negative otherwise. As predictors, this model now included the expected variability at current trial, the observed variability at current trial, the observed variability at previous trial, and the outcome PE at previous trial (i.e., the distance between the number chosen by participants and at previous trial and the subsequent outcome revealed). In line with the model discussed in the main text, regression coefficients for expected and observed variability at current trial were significantly positive across participants (t(32) = 11.95, p < 0.001 and t(32) = 3.59, p = 0.001, respectively). The coefficient for observed variability at previous trial was not significant (t(32) = 62, p = 0.536), while the coefficient for outcome PE was significant (t(32) = 2.85, p = 0.008). The latter result indicates that participants’ response was closer to the expected value after a trial characterized by a large discrepancy between response and the outcome revealed.

**Analyses of stability of model-based behavioural effects**

We asked whether the behavioural effects derived from optimal inference theory and tested in the main text were stable along the task. To do this, we asked whether these effects changed when comparing the first half versus the second half of the task. Specifically:

- For *Prediction one* (according to which $\bar{v}_{H}$ should be larger than $\bar{v}_{L}$) we ran an ANOVA with session (first vs second half) and parameter ($\bar{v}_{H}$ vs $\bar{v}_{L}$) as factors. Consistent with predictions (see also the main text), we found a main effect of parameter (F(1,32) = 35, p < 0.001). No main effect of session (F(1,32) = 0.021, p = 0.885) emerged. Crucially, we found no evidence of any interaction (F(1,32) = 0.069, p = 0.795), indicating that the effect of parameter was stable across sessions.
- For *Prediction two* (according to which $a_{g}$ should be larger than zero), we compared the parameter across sessions and no difference emerged (t(32) = 0.59, p = 0.561). This shows that the effect related with this parameter was stable across sessions.
- For *Prediction three* (implying that the value of $\bar{\omega}_{H}$ would be larger than the value of $\bar{\omega}_{L}$) we ran an ANOVA with session (first vs second half) and parameter ($\bar{\omega}_{H}$ vs$\bar{\omega}_{L}$) as factors. Consistent with predictions (see also the main text), we found a main effect of parameter (F(1,32) = 22.30, p < 0.001). A main effect of session (F(1,32) = 12.48, p = 0.001) also emerged, indicating that stochasticity was overall higher in the second session. Note that this finding does not undermine the claim that prediction three is valid for both sessions. The crucial test for this claim is the interaction, and results showed this to be non-significant (F(1,32) = 0.004, p = 0.951). This result indicates that the effect of parameter is stable across sessions.
- For *Prediction four* (according to which $b_{g}$ should be larger than zero), we compared the parameter across sessions and no difference emerged (t(32) = 0.43, p = 0.670). This shows that the effect related with this parameter is stable across sessions.

**Analyses of synthetic data**

We performed a control analysis to assess whether the full model was necessary to account for the results reported above regarding our four predictions. In other words, it is important to ascertain that each behavioural effect is directly produced by the appropriate component of the model, and it is not the indirect consequence of other components. We used the full model and the simpler models to generate synthetic data (using the empirical model parameter estimates), and asked whether analysis of synthetic data would lead to the same conclusions. Finding that a simpler model could reproduce our results would cast doubts on whether the full model was necessary. Each model was used to estimate the parameters of each participant, and based on these parameters a synthetic dataset was simulated. From the simulated dataset, we estimated parameters (under the full model) and repeated the statistical tests of our four predictions. This was replicated 1000 times per model, and the average t-value was computed. Using the full model to simulate the data, all four results were replicated (fig. S3; $\bar{v}_{H}>\bar{v}_{L}:$ t(32) = 5.48, p < 0.001;$a_{g}>0$: t(32) = 2.36, p = 0.024; $\bar{\omega}_{L}>\bar{\omega}_{H}$: t(32) = 4.52, p < 0.001; $b_{g}>0$: t(32) = 6.18, p < 0.001). When ${Model}_{\bar{v}}$ was used to simulate the data, all effects were obtained except the difference between $\bar{v}_{H}$ and $\bar{v}_{L}$ (t(32) = 0.63, p = 0.535). When data were simulated with ${Model}_{a}$, all effects were obtained except for a $a_{g}>0$ (t(32) = 0.85, p = 0.402). The difference between $\bar{\omega}_{L}$ and $\bar{\omega}_{H}$ was not replicated when ${Model}_{\omega}$ was used to simulate the data (t(32) = 0.28, p = 0.779), while the remaining effects were replicated. When data were simulated with ${Model}_{b}$, all effects were replicated except for a $b_{g}>0$ (t(32) = -1.08, p = 0.289). These synthetic results show that the full model, but not simpler models, was able to account for all behavioural results, suggesting that all components of the model are necessary. In addition, a link between each element of the model and a specific behavioural effect emerged; reflecting the fact that removing a specific element of the model compromised its ability to reproduce a specific effect.

|  | $\boldsymbol{EV}_{\boldsymbol{H}}$ | $\boldsymbol{PE}_{\boldsymbol{H}}$ | $\boldsymbol{OV}_{\boldsymbol{H}}$ | $\boldsymbol{RT}_{\boldsymbol{H}}$ | $\boldsymbol{EV}_{\boldsymbol{L}}$ | $\boldsymbol{PE}_{\boldsymbol{L}}$ | $\boldsymbol{OV}_{\boldsymbol{L}}$ | $\boldsymbol{RT}_{\boldsymbol{L}}$ | **F** | $\boldsymbol{PE}_{\boldsymbol{F}}$ | **R** |
| --- | --- | --- | --- | --- | --- | --- | --- | --- | --- | --- | --- |
| $\boldsymbol{EV}_{\boldsymbol{H}}$ |  |  |  |  |  |  |  |  |  |  |  |
| $\boldsymbol{PE}_{\boldsymbol{H}}$ | $\bar{c}=.02$  $c_{M}$=.11 |  |  |  |  |  |  |  |  |  |  |
| $\boldsymbol{OV}_{\boldsymbol{H}}$ | $\bar{c}=.01$  $c_{M}$=.13 | $\bar{c}=.04$  $c_{M}$=.21 |  |  |  |  |  |  |  |  |  |
| $\boldsymbol{RT}_{\boldsymbol{H}}$ | $\bar{c}=.02$  $c_{M}$=.09 | $\bar{c}=.03$  $c_{M}$=.11 | $\bar{c}=.02$  $c_{M}$=.10 |  |  |  |  |  |  |  |  |
| $\boldsymbol{EV}_{\boldsymbol{L}}$ | $\bar{c}=.03$  $c_{M}$=.15 | $\bar{c}=.03$  $c_{M}$=.09 | $\bar{c}=.02$  $c_{M}$=.11 | $\bar{c}=.02$  $c_{M}$=.18 |  |  |  |  |  |  |  |
| $\boldsymbol{PE}_{\boldsymbol{L}}$ | $\bar{c}=.03$  $c_{M}$=.17 | $\bar{c}=.01$  $c_{M}$=.13 | $\bar{c}=.03$  $c_{M}$=.13 | $\bar{c}=.03$  $c_{M}$=.27 | $\bar{c}=.03$  $c_{M}$=.13 |  |  |  |  |  |  |
| $\boldsymbol{OV}_{\boldsymbol{L}}$ | $\bar{c}=.02$  $c_{M}$=.08 | $\bar{c}=.03$  $c_{M}$=.12 | $\bar{c}=.03$  $c_{M}$=.15 | $\bar{c}=.01$  $c_{M}$=.19 | $\bar{c}=.03$  $c_{M}$=.14 | $\bar{c}=.01$  $c_{M}$=.15 |  |  |  |  |  |
| $\boldsymbol{RT}_{\boldsymbol{L}}$ | $\bar{c}=.02$  $c_{M}$=.08 | $\bar{c}=.01$  $c_{M}$=.11 | $\bar{c}=.02$  $c_{M}$=.18 | $\bar{c}=.01$  $c_{M}$=.18 | $\bar{c}=.02$  $c_{M}$=.11 | $\bar{c}=.01$  $c_{M}$=.16 | $\bar{c}=.03$  $c_{M}$=.14 |  |  |  |  |
| **F** | $\bar{c}=.56$  $c_{M}$=.68 | $\bar{c}=.01$  $c_{M}$=.11 | $\bar{c}=.02$  $c_{M}$=.11 | $\bar{c}=.01$  $c_{M}$=.19 | $\bar{c}=.61$  $c_{M}$=.71 | $\bar{c}=.02$  $c_{M}$=.20 | $\bar{c}=.04$  $c_{M}$=.11 | $\bar{c}=.02$  $c_{M}$=.11 |  |  |  |
| $\boldsymbol{PE}_{\boldsymbol{F}}$ | $\bar{c}=.02$  $c_{M}$=.15 | $\bar{c}=.02$  $c_{M}$=.14 | $\bar{c}=.03$  $c_{M}$=.17 | $\bar{c}=.02$  $c_{M}$=.11 | $\bar{c}=.02$  $c_{M}$=.11 | $\bar{c}=.02$  $c_{M}$=.15 | $\bar{c}=.01$  $c_{M}$=.13 | $\bar{c}=.01$  $c_{M}$=.18 | $\bar{c}=.02$  $c_{M}$=.21 |  |  |
| **R** | $\bar{c}=.02$  $c_{M}$=.11 | $\bar{c}=.03$  $c_{M}$=.15 | $\bar{c}=.01$  $c_{M}$=.17 | $\bar{c}=.02$  $c_{M}$=.13 | $\bar{c}=.02$  $c_{M}$=.11 | $\bar{c}=.03$  $c_{M}$=.15 | $\bar{c}=.01$  $c_{M}$=.12 | $\bar{c}=.02$  $c_{M}$=.19 | $\bar{c}=.03$  $c_{M}$=.21 | $\bar{c}=.01$  $c_{M}$=.11 |  |

**Tab. S1**. Matrix assessing collinearity among the regressors included in the main GLM. Each cell reports a statistic quantifying collinearity between two regressors averaged across participants ($\bar{c}$) and for the participant showing the strongest value for the statistic for those regressors ($c_{M};$ i.e, |c|). The statistics used to quantify collinearity is c = cos(θ), which is provided by the software SPM. Regressors considered in the matrix are ${EV}_{H}$: stick function regressor for high expected variability; ${PE}_{H}$: its associated PE; ${OV}_{H}:$ its associated observed variability; ${RT}_{H}$: its associated RTs; ${EV}_{L}$: stick function regressor for low expected variability; ${PE}_{L}$: its associated PE; ${OV}_{L}:$ its associated observed variability; ${RT}_{L}$: its associated RTs; F: stick function regressor for feedback; ${PE}_{F}$: its associated PE; R: a box-car function regressor capturing response duration. The design matrix shows no issue due to collinearity among regressors of interest. Please note that high collinearity is evident for the regressor F and ${EV}_{H}$ and for the regressor F and ${EV}_{L}$, which do not affect our analyses. Information about collinearity involving movement and physiological regressors is not reported in the matrix. The average statistic for these regressors was $\bar{c}=0.06$ and the strongest statistic was $c_{M}=0.21$.

High minus low expected variability

| **Area** | **Coordinates** | **T value** |
| --- | --- | --- |
| Posterior superior parietal lobule left | -9, -52, 55 | 4.42 |
| Posterior superior parietal lobule right | 6, -52, 52 | 4.74 |
| Right angular gyrus | 45, -67, 34 | 5.53 |
| Temporoparietal junction right | 39, -55, 22 | 5.69 |
| Posterior superior temporal lobe right | 54, -25, 13 | 3.82 |
| Lateral temporal lobe left | -66, -7, -2 | 4.11 |
| Posterior dorsolateral prefrontal cortex right | 24, 29, 49 | 4.49 |
| Anterior dorsolateral prefrontal cortex right | 27, 38, 34 | 4.10 |
| Anterior dorsolateral prefrontal cortex rleft | -39, 38, 22 | 4.72 |

Positive relationship with observed variability

| **Area** | **Coordinates** | **T value** |
| --- | --- | --- |
| Posterior insula right | 36, -25, 25 | 4.46 |
| Posterior insula left | -30, -13, 28 | 3.92 |
| Lateral temporal lobe left | -45, -4, 22 | 4.23 |

Negative relationship with observed variability

| **Area** | **Coordinates** | **T value** |
| --- | --- | --- |
| Posterior lateral parietal cortex left | -48, -58, 46 | 4.26 |
| Anterior cingulate cortex right | 9, 38, 37 | 4.25 |

Positive relationship with PE

| **Area** | **Coordinates** | **T value** |
| --- | --- | --- |
| Inferior occipital cortex right | 18, -97, -2 | 3.68 |
| Inferior occipital cortex left | -18, -100, -2 | 3.55 |
| Superior frontal gyrus right | 21, 5, 55 | 4.54 |
| Superior frontal gyrus left | -27, 2, 64 | 3.73 |

Negative relationship with PE

| **Area** | **Coordinates** | **T value** |
| --- | --- | --- |
| Superior occipital cortex left | -6, -91, 28 | 3.57 |
| Dorsomedial prefrontal cortex | 0, 50, 43 | 3.53 |

Positive relationship with outcome PE (i.e., at the time of feedback)

| **Area** | **Coordinates** | **T value** |
| --- | --- | --- |
| Left posterior cingulate | -18 -61 19 | 4.91 |
| Right posterior cingulate | 15 -55 19 | 7.52 |
| Left parahippocampal gyrus | -27 -64 -5 | 5.9 |
| Right parahippocampal gyrus | 24 -58 -5 | 4.23 |
| Left angular gyrus | -30 -79 34 | 5.22 |
| Right angular gyrus | 42 -82 34 | 5.23 |

Negative relationship with outcome PE (i.e., at the time of feedback)

| **Area** | **Coordinates** | **T value** |
| --- | --- | --- |
| Left orbitofrontal cortex | -27 47 -14 | 5.98 |
| Ventromedial prefrontal cortex | 6 44 -5 | 5.41 |
| Medial prefrontal cortex | -3 44 19 | 7.48 |
| Ventral tegmental area | -6 -16 -14 | 6.78 |
| Left ventral striatum | -12 11 -2 | 10.14 |
| Right ventral striatum | 9 11 -5 | 10.00 |
| Left central sulcus | -60 -19 28 | 7.62 |
| Central cingulate cortex | -3 -22 37 | 6.19 |
| Left supramarginal gyrus | -60 -43 43 | 6.29 |
| Right supramarginal gyrus | 57 -43 34 | 6.76 |
| Vermis | 3 -61 -26 | 5.07 |
| Left occipital cortex | -30 -91 -5 | 4.45 |
| Right occipital cortex | 36 -88 -2 | 5.55 |

**Table S2**. Analysis of that brain volume extending outside our ROI space (remember our ROIs were hippocampus and SPC). For the different contrasts, we report voxels within this volume that have statistics associated with p<0.001 uncorrected (no voxel satisfied this criterion for the contrast comparing low minus high expected variability).

**
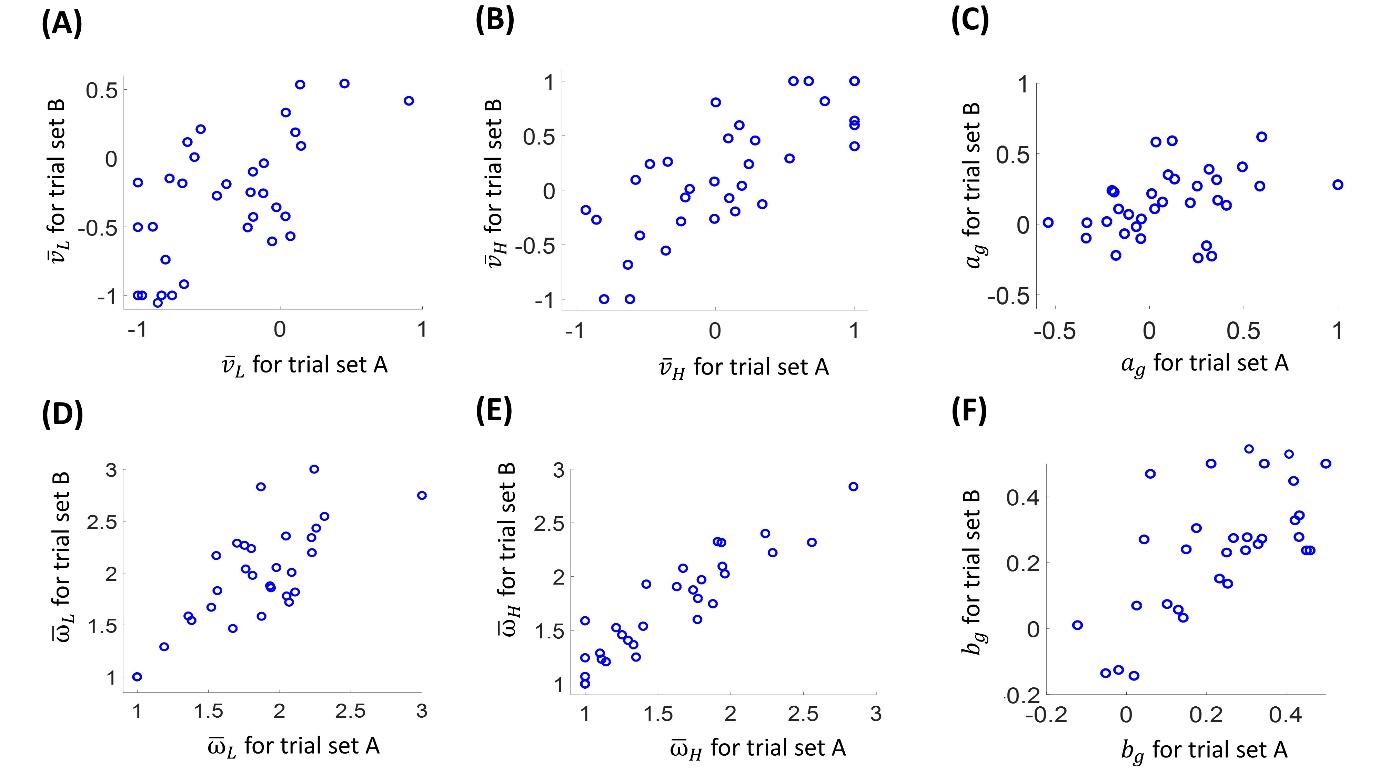
**

**Figure S1**. Reliability analysis of the parameters estimated with the full model. For each participant, trials were randomly split in two sets (A and B), and the parameters were estimated separately for each set. Relationship between trial set A and B relative to **A**: the parameter indicating trials with low expected variability $\bar{v}_{L}$ (r(31) = 0.63, p < 0.001); **B**: the parameter indicating trials with high expected variability $\bar{v}_{H}$ (r(31) = 0.77, p < 0.001); **C**: the parameter $a_{g}$ which captures the effect of the z-scored observed variability $v_{g}^{'}$ (r(31) = 0.39, p = 0.026); **D**: the parameter $\bar{\omega}_{L}$ reflecting an effect of trials with low expected variability on the response stochasticity (r(31) = 0.82, p < 0.001); **E**: the parameter $\bar{\omega}_{H}$ reflecting an effect of trials with high expected variability on the response stochasticity (r(31) = 0.93, p < 0.001); **F**: the parameter $b_{g}$ which captures the effect of the z-scored observed variability $v_{g}^{'}$ on stochasticity (r(31) = 0.60, p < 0.001).


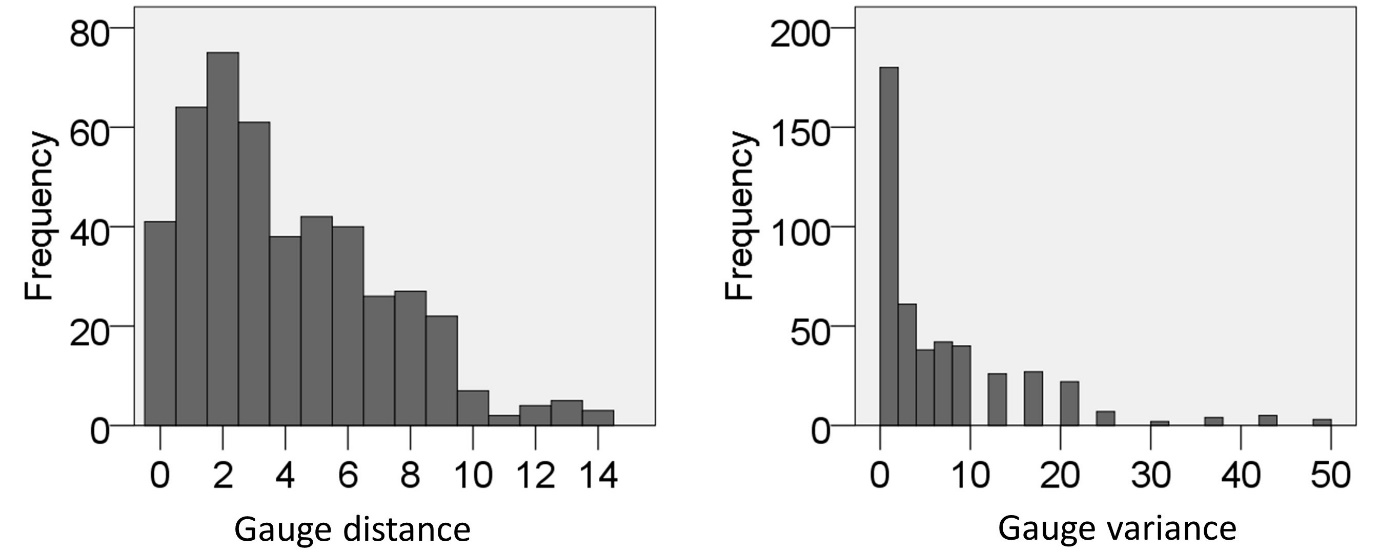


**Figure S2**. Frequency distribution across trials of gauge distance ($v_{g}=|g_{1}$ - $g_{2}|$), on the left, and gauge variance (${VAR}_{g}=\frac{\sum{(g_{x}-\mu_{g})}^{2}}{2}$), on the right. Data are relative to one single participant. Remember that $g_{1}$ and $g_{2}$ indicate the two numbers reported by the gauges and $\mu_{g}$ indicates their average (i.e., $\mu_{g}=(g_{1}+g_{2})/2$).

**
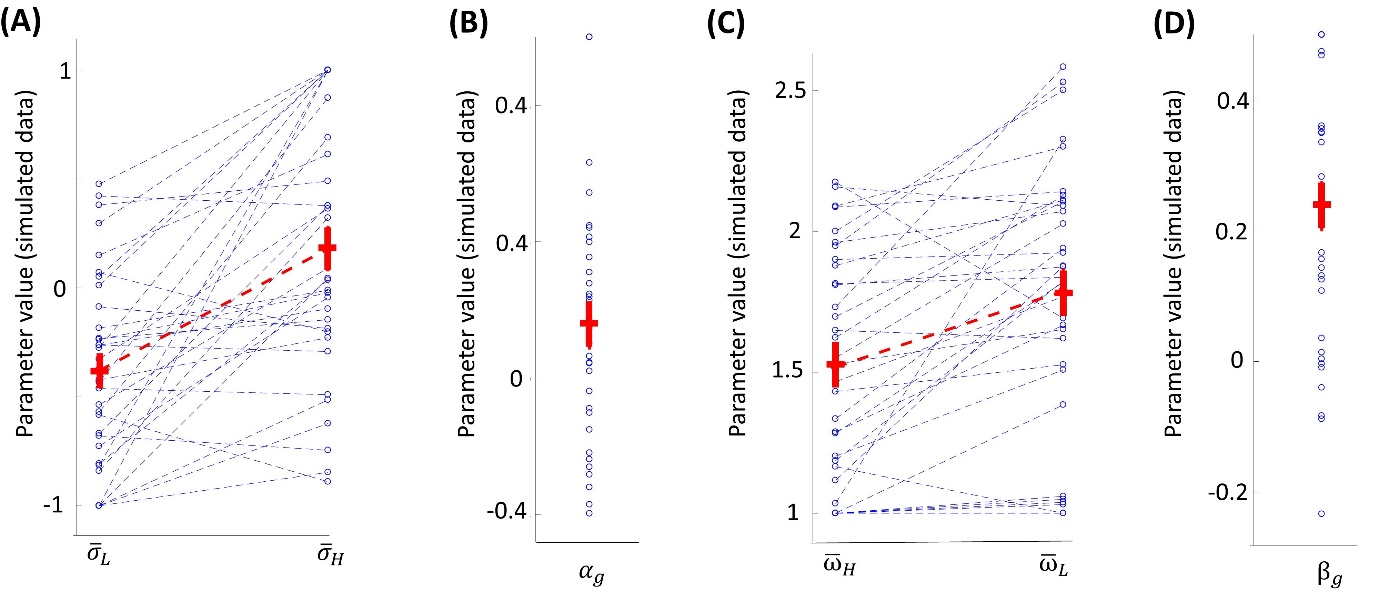
**

**Figure S3**. Test of the predictions derived from the optimal inference hypothesis performed on data simulated with the full generative model of behaviour. Horizontal red lines indicate averages across participants, vertical red lines indicate the standard error, and blue dots indicate values for individual participants. **A**: Prediction one implying that $\bar{v}_{H}>\bar{v}_{L}$ (t(32) = 5.48, p < 0.001). **B**: Prediction two implying that$a_{g}>0$ (t(32) = 2.36, p = 0.024). **C**: Prediction three implying that $\bar{\omega}_{L}>\bar{\omega}_{H}$ (t(32) = 4.52, p < 0.001). **D**: Prediction four implying that $b_{g}>0$ (t(32) = 6.18, p < 0.001).
